# 2199. Antibiotic tracking and reporting in skilled nursing facilities: a failure to communicate

**DOI:** 10.1093/ofid/ofad500.1821

**Published:** 2023-11-27

**Authors:** Sally Jolles, Dee Heller, Lindsay Taylor, Ashlie Dowdell, Christopher J Crnich, Jay Ford

**Affiliations:** University of Wisconsin School of Medicine and Public Health, Madison, Wisconsin; University of Wisconsin-Madison, Madison, Wisconsin; University of Wisconsin School of Medicine and Public Health, Madison, Wisconsin; Wisconsin Division of Public Health, Madison, WI; University of Wisconsin School of Medicine and Public Health, Madison, Wisconsin; UW Madison School of Pharmacy, Madison, Wisconsin

## Abstract

**Background:**

Antibiotic overuse and misuse are common in skilled nursing facilities (SNFs) and are drivers of adverse drug events, antibiotic resistance and Clostridioides difficile infections. CMS requires SNFs to implement antibiotic stewardship programs (ASPs) that include a system for tracking and reporting antibiotic use (AU) and antibiotic-related outcome (ARO) measures. However, there is limited understanding of the maturity of these systems in SNFs.

**Methods:**

We conducted a descriptive study of AU/ARO tracking and reporting practices in 10 Wisconsin SNFs recruited in the fall of 2022. Key informants in study SNFs were asked to provide de-identified examples of line lists and facility antibiotic reports that included AU/ARO information. Checklists were developed to identify the types and characteristics of the different AU/ARO variables and measures. Document analyses were conducted on the retrieved source documents utilizing these checklists.

**Results:**

36 source documents were analyzed including 24 line lists and 12 AU reports (Figure 1). Of the 20 qualifying line lists, 40% were in a format that permitted direct data manipulation (e.g., Excel spreadsheet). 70% of SNFs employed multiple line lists. 30% of SNFs were unable to provide an aggregate AU report. Although a majority of SNF line lists captured information on antibiotic indication, duration of therapy and ordering prescriber, these variables were frequently omitted from facility AU reports (Figure 2). Information on appropriateness of antibiotic prescriptions and origin of the antibiotic order (e.g., ED) were captured less frequently on SNF line lists and, by extension, facility AU reports (Figure 2).

**Figure:**
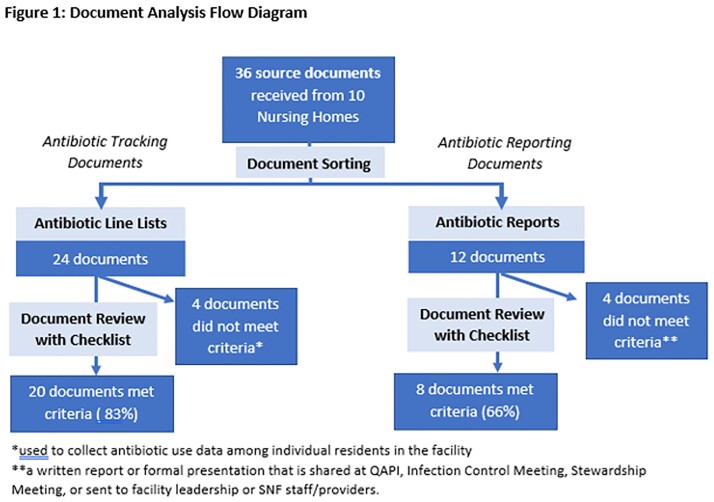

A comparison of facility Line Lists versus Reports

**Figure:**
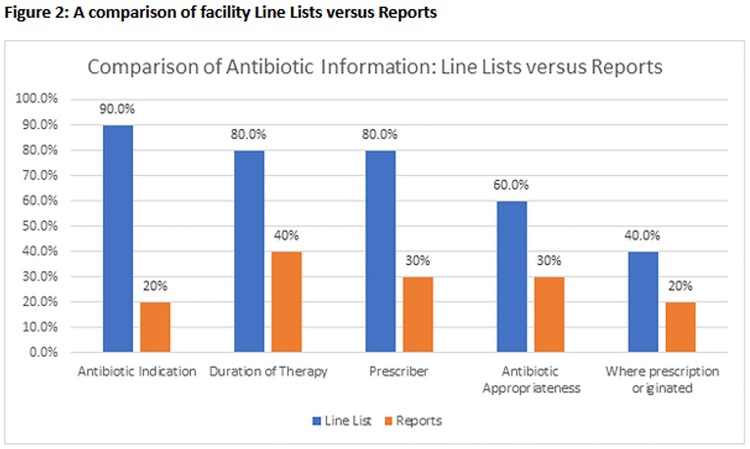

**Conclusion:**

SNF staff devote considerable resources to identifying and characterizing antibiotic events. However, this information is not stored in a format that allows data to be reviewed and analyzed easily. Additionally, a significant minority of SNFs do not routinely generate AU/ARO reports and those that do, often omit important information about prescribing practices that could inform facility quality improvement efforts. These findings suggest there is substantial opportunity to improve SNF AU/ARO tracking and reporting practices.

**Disclosures:**

**Lindsay Taylor, MD, MS**, Merck: Grant/Research Support

